# Reusable and pH-Stable Luminescent Sensors for Highly Selective Detection of Phosphate

**DOI:** 10.3390/polym14010190

**Published:** 2022-01-04

**Authors:** Do Yeob Kim, Dong Gyu Kim, Bongjin Jeong, Young Il Kim, Jungseok Heo, Hyung-Kun Lee

**Affiliations:** 1 ICT Creative Research Laboratory, Electronics & Telecommunications Research Institute, Daejeon 34129, Korea; nanodykim@etri.re.kr (D.Y.K.); jbj0919@etri.re.kr (B.J.); 2 Department of Chemistry, Chungnam National University, Daejeon 34134, Korea; kdg05262@naver.com (D.G.K.); yi4902@naver.com (Y.I.K.)

**Keywords:** coordination polymer particle, luminescence, quenching, phosphate sensor, environmental analysis

## Abstract

Phosphate sensors have been actively studied owing to their importance in water environment monitoring because phosphate is one of the nutrients that result in algal blooms. As with other nutrients, seamless monitoring of phosphate is important for understanding and evaluating eutrophication. However, field-deployable phosphate sensors have not been well developed yet due to the chemical characteristics of phosphate. In this paper, we report on a luminescent coordination polymer particle (CPP) that can respond selectively and sensitively to a phosphate ion against other ions in an aquatic ecosystem. The CPPs with an average size of 88.1 ± 12.2 nm are embedded into membranes for reusable purpose. Due to the specific binding of phosphates to europium ions, the luminescence quenching behavior of CPPs embedded into membranes shows a linear relationship with phosphate concentrations (3–500 μM) and detection limit of 1.52 μM. Consistent luminescence signals were also observed during repeated measurements in the pH range of 3–10. Moreover, the practical application was confirmed by sensing phosphate in actual environmental samples such as tap water and lake water.

## 1. Introduction

Algal blooms are known to occur in lakes, rivers, and oceans, with complex behavior due to various prevailing factors such as nutrients (NO_3_^−^, NH_4_^+^, and PO_4_^3−^) along with water temperature, flow rate, rainfall, and dissolved oxygen [1]. In particular, harmful algal blooms (HABs) of *Microcystis*, *Anabaena*, *Oscillatoria*, and *Aphanizomenon* produce toxins and have a malignant effect on aquatic life and humans [2,3]. Extensive research has been conducted on the conservation of aquatic environments through seamless data monitoring of HABs, prediction of their occurrence, and intervention of appropriate algae-removal treatments [4]. To this end, water environments have been monitored using various sensors such as pH, chlorophyll a, phycocyanin, total nitrogen, NH_4_-N, NO_3_-N, total phosphorous (TP), and PO_4_-P sensors. Most sensors can be deployed in the field and applied seamlessly to gather signals using an Internet-of-Things platform. However, in the case of TP and PO_4_-P, there is a general method for quantifying the reaction with a coloring agent after reacting with an oxidizing agent under the conditions of high temperature and high pressure. This complicated analysis process limits the field deployment applications of phosphate sensors without the aid of chemical reagents and makes phosphate sensors only applicable in the laboratory. Phosphate sensors have been proposed and devised for working with various mechanisms such as solid electrochemical redox types [5], liquid membrane-ion selective electrodes (ISE) [6], solid-state ISE [7,8], spectrophotometric, and photoluminescence types [9,10,11]. However, there are several difficulties in developing phosphate sensors. Firstly, phosphate ions prefer different forms according to the acidity of the dissolved aqueous solution. This makes it difficult to realize phosphate sensors that can work in a wide pH range. Secondly, the widely adapted size-exclusion principle for sensor selectivity is difficult to apply in phosphate sensors owing to its relatively large ion size. Furthermore, it is hard to implement a phosphate sensor using non-covalent interactions such as hydrogen bonding as it has a large solvation energy according to the *Hofmeister* series [12]. Although ISE sensors have been studied for more than 45 years and show high performance for many cations and anions (NH_4_^+^, K^+^, Na^+^, Mg^2+^, Ca^2+^, Cl^−^, NO_3_^−^, CN^−^, and F^−^) [13], no ISE-type phosphate sensor with sufficient reliability in field applications has been developed. Note that it is necessary to detect the concentration of PO_4_-P at the level of 0.1 mg/L (3.2 μM), being an indicative level for probable problematic algal growth [14]. This detection level of phosphate is accomplished using spectrophotometric or photoluminescent analysis in an experimental setup consisting of complex fluid control devices and chemical storage/supplies [15].

**Figure 1:**
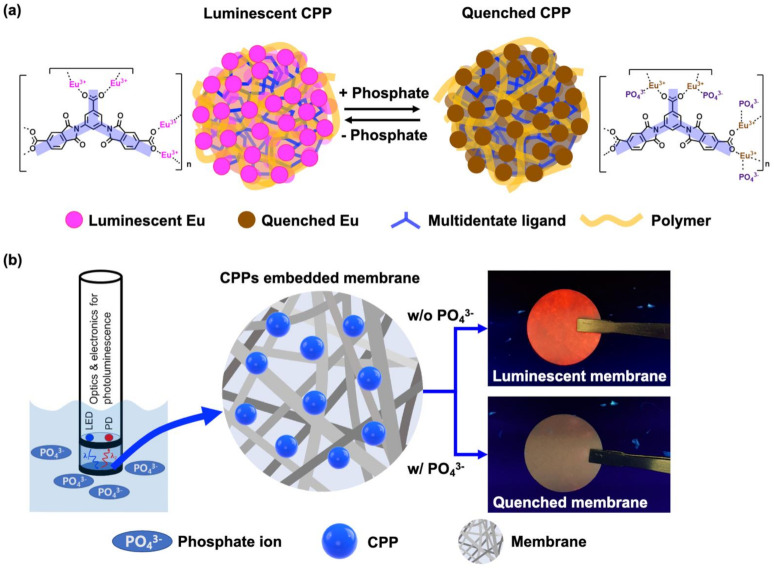
(**a**) Schematic illustration of the luminescent “turn-off” sensing based on CPP for detection of phosphate. (**b**) Conceptual configuration of phosphate sensor composed of light source, detector, and membrane containing CPPs as sensing materials.

Research areas on coordination polymer particles (CPPs) are expanding rapidly to various applications such as gas storage, catalysis, molecular sensing, bio-imaging, and drug delivery due to their significantly large surface area and tunability [16,17]. Our research group has made many efforts to develop diverse CPPs by using rationally designed functional building blocks [18,19]. Surface modification and control of size and shape over CPPs are considered critical for practical applications because a subtle change in the interface alters their physical and chemical properties [20]. Recently, several investigations have been also reported on the applications of phosphate sensors based on metal–organic frameworks [9,21,22,23,24]. In this study, to develop a phosphate sensor that can be easily deployed in the field and can be operated unattended, we present a photoluminescence phosphate sensor based on an CPP with high sensitivity, selectivity, and wide working range of pH. The strong coordination interaction between europium and phosphate in the CPP is considered to provide exceptional selectivity and sensitivity as well as stability in various pH (Figure 1a). The CPP in this study is more durable in the aquatic environment than common luminescent metal-organic frameworks and can be synthesized in the form of nanoparticles that can be easily embedded into the porous membrane [25,26]. The resulting luminescent membrane containing the CPP is expected to be installed in a probe-type photoluminescent sensor adapted for the commercial dissolved-oxygen sensor as shown in Figure 1b [27].

## 2. Materials and Methods

### 2.1. Preparation of Luminescent CPP

EuCl_3_∙6H_2_O was purchased from TCI (Tokyo, Japan), and polyvinylpyrrolidone (PVP) was bought from Thermo Scientific^TM^ (Massachusetts, USA). Other reagents such as dimethylformamide (DMF), ethanol, potassium phosphate and H_2_SO_4_ were purchased from Samchun Chemicals (Pyeongtaek, Korea). 2,2′-(5-Carboxy-1,3-phenylene)bis(1,3-dioxoisoindoline-5-carboxylic acid) (TCA) was synthesized according to a previously reported method with modification [28]. Eu-TCAs composed of Eu ions and TCA as a multidentate ligand containing three carboxylic groups were synthesized in the form of CPP under solvothermal and high-pressure conditions. EuCl_3_∙6H_2_O (235 mg, 0.64 mmol, 5.3 equation), TCA (60 mg, 0.12 mmol, 1 equation), and PVP (Avg. Mol. Wt. = 50,000, 1600 mg) were mixed in DMF (32 mL) and ethanol (19 mL). The reaction mixture was acidified by adding H_2_SO_4_ (33.3 μL) and then sonicating for 30 min to obtain a clear solution. A hydrothermal reactor was charged with the reaction mixture, and a solvothermal reaction condition was applied at 150 °C for 12 h. The reaction mixture was cooled over the next 6 h, and colloidal particles were precipitated by centrifugation. Three cycles of wash with dry DMF and centrifugation were followed by a final wash with ethanol. After drying at 70 °C for 3 h, a pinkish-white solid was obtained. 

### 2.2. Preparation of Eu-TCA Embedded in Glass Microfiber Filters (Eu-TCA/GMF)

GMFs with a pore size of 1.2 μm were used as a membrane. First, the GMF was placed at the bottom of each well of a 96-well microplate. An Eu-TCA dispersion with a concentration of 0.5 wt.% was prepared and sonicated for 10 min. Next, each well was filled with 60 μL of the Eu-TCA dispersion and dried overnight. The microplate containing the Eu-TCA/GMF was washed with deionized water several times to remove unadsorbed Eu-TCA particles to the GMF.

### 2.3. Phosphate Detection of Eu-TCA Dispersion

In the study of the quenching of Eu-TCA dispersion by phosphate, 10 mg of Eu-TCA powder was dispersed in 25 mL of deionized water. After ultrasonication for 20 min, 2.5 mL of Eu-TCA stock solution and the different concentrations of phosphate solution (2.5 mL) were added to a test tube. The solution was thoroughly mixed and then incubated for 10 min before photoluminescence measurements.

### 2.4. Characterization

The surface morphology and microstructure of the Eu-TCA were analyzed using field-emission scanning electron microscopy (SEM, S-4800, Hitachi, Tokyo, Japan) and transmission electron microscopy (TEM, Tecnai G2 F30, FEI, USA) with energy dispersive X-ray spectroscopy (EDX). Powder X-ray diffraction (PXRD) data were collected using a Rigaku diffractometer (MiniFlex II, Rigaku, Japan). The diffraction pattern was measured with Cu-Kα radiation (λ = 1.5418 Å) with voltage of 30 kV and current of 15 mA. The 2θ range was 5–50°. Fourier transform infrared (FTIR) spectra were obtained using a Nicolet iS10 FTIR spectrometer (Thermo Fisher Scientific, Waltham, MA, USA), and X-ray photoelectron spectroscopy (XPS) measurements were performed by using a K-Alpha^+^ spectrometer (Thermo Fisher Scientific, USA) equipped with a micro-focused monochromatic Al Kα X-ray source. The photoluminescence of the Eu-TCA dispersion was studied using a spectrophotometer (FL 6500, PerkinElmer, Waltham, MA, USA). To investigate the phosphate sensing performance of the Eu-TCA/GMF, the luminescence intensity of the microplate containing the Eu-TCA/GMF was measured by using a microplate reader (Infinite M200, TECAN, Swiss, Basel, Switzerland). The excitation and emission wavelengths were selected as 260 and 615 nm, respectively.

## 3. Results and Discussion

### 3.1. Morphology and Structure Analysis

The Eu-TCA was successfully obtained by controlling the synthetic parameters: the molar ratio between Eu^3+^ ions and ligands, concentration, reaction temperature, and pH. PVP addition and pH control affected the shape and crystallinity of CPPs. PVP contains pyrrolidone groups along their backbone, and the carbonyl groups on pyrrolidones weakly bind to metal ions. These additional coordination bonds interrupt the bonds between Eu^3+^ ions and TCA ligands, leading to an irregular arrangement of components around the surface region. They also play an important role in controlling the size of particles. TCA ligand molecules and metal ions concentrate around the PVP molecule even in the early state and at lower pH conditions. For this reaction, an appropriate amount of PVP relative to the amounts of Eu ions and TCA ligands should be used to achieve size and shape control. When the amount of PVP was reduced to one-third of the synthetic condition as described in Materials and Methods, the Eu-TCA was obtained as anisotropic rice-shaped particles (Supplementary Figure S1).

It is important to prepare homogeneous CPP dispersion in that it ensures batch-to-batch reproducibility and thus provides reliable detection of phosphate. Size control was improved by adding sulfuric acid to the reaction mixture. This was used to delay the rate of pH elevation owing to decomposition of DMF into dimethyl amine. Kinetic control over CPP formation requires such pH adjustment at the early stage of the reaction or temperature control over the reaction time [29].

**Figure 2:**
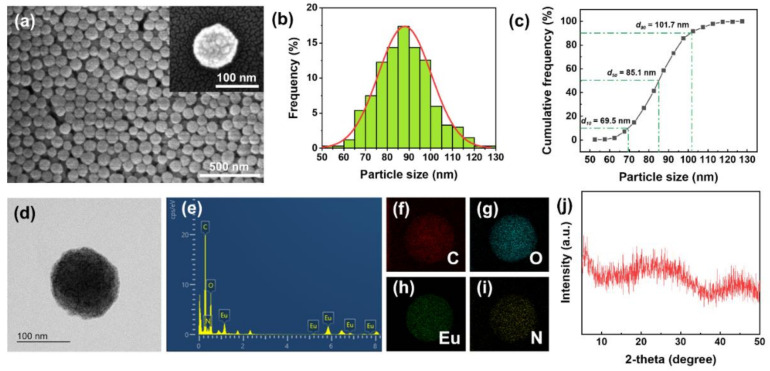
(**a**) SEM images, (**b**) size histogram, (**c**) cumulative frequency distribution, (**d**) TEM image, (**e**) EDX spectrum, (**f**–**i**) EDX elemental mapping, and (**j**) XRD spectrum of Eu-TCA.

The morphology of the Eu-TCA was studied by SEM and TEM analysis. As shown in Figure 2a, the obtained Eu-TCA particles are spherical. The size distribution of the Eu-TCA was determined based on the SEM image by measuring the size of 334 particles. It was confirmed that the Eu-TCA exhibits normal distribution with an average size of 88.1 ± 12.2 nm (Figure 2b). In addition, as shown in Figure 2c, the cumulative frequency distribution indicates that most of the Eu-TCAs are less than 101.7 nm (*d*_90_). The morphology and size of the Eu-TCA from TEM observations were in good agreement with those in the SEM analysis and dynamic light scattering measurement (Figure 2d and Supplementary Figure S2). The elemental composition of the CPPs was estimated by using EDX. As shown in Figure 2e, Eu, C, and O peaks appear as the main strong peaks, whereas N is a minor peak. This result indicated that a large portion of the CPP is a contribution of Eu^3+^ ions, and the remaining is shared by TCA ligands and PVP polymers. It is clear from the elemental mapping of the single Eu-TCA that C, O, Eu, and N are uniformly distributed throughout the spherical particle (Figure 2f–i). PXRD measurements of the particles exhibited no strong diffraction peaks (Figure 2j). These results indicated the formation of amorphous coordination polymers due to the premature short-range regularity.

### 3.2. Evaluation of Phosphate Sensing Materials Based on Luminescence Quenching

**Figure 3:**
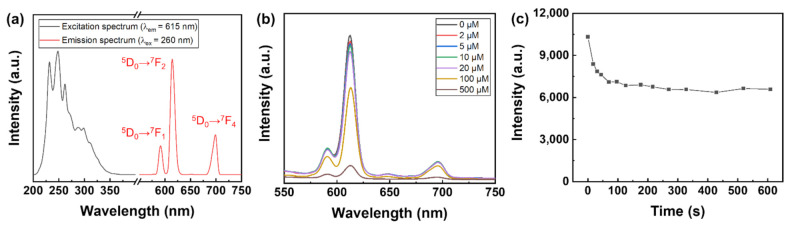
(**a**) Photoluminescence spectra of Eu-TCA dispersion. (**b**) Luminescence spectra of Eu-TCA dispersion in the presence of different amount of phosphate under the excitation at 260 nm. (**c**) Time-dependent luminescence intensity of Eu-TCA dispersion upon the addition of 100 μM of phosphate.

Figure 3a shows the excitation and emission spectra of the Eu-TCA dispersion in deionized water (200 mg/L). The excitation spectrum was measured at an emission wavelength of 615 nm, and the emission spectrum was determined under excitation at 260 nm. The broad band in the range of 230–300 nm in the excitation spectrum results from the O^2−^ → Eu^3+^ charge transfer and π–π* electronic transition in coordinated organic ligands [30,31,32]. As shown in Figure 3a and Supplementary Figure S3, the emission spectra exhibit five characteristic transitions of Eu^3+^ centered at 579, 591, 615, 651, and 699 nm, corresponding to the ^5^D_0_ → ^7^F*_J_* transitions (*J* = 0, 1, 2, 3, and 4), respectively [33].

To investigate the detection performance of the Eu-TCA for phosphate, luminescence titration experiments were conducted at various concentrations of phosphate. Luminescence quenching occurred upon the addition of phosphate, and the luminescence intensity decreased with the increase in phosphate concentration (Figure 3b). The efficiency of the luminescence quenching process can be determined by the following Stern–Volmer equation:

(1) $$I_{0}/I=1+K_{SV}\times \left[Q\right],$$

where *I* and *I_0_* are the luminescence intensities with and without the quencher, respectively, *K*_SV_ is the quenching constant, and [*Q*] is the molar concentration of the quencher. Supplementary Figure S4 shows the Stern–Volmer plot for the ^5^D_0_ → ^7^F_2_ transition at 615 nm for the Eu-TCA dispersion. A good linear Stern–Volmer relationship between the ratio of luminescence intensity (*I*_0_/*I*) and concentrations ranging from 2 to 100 μM was observed with a *K*_SV_ of 5.78 × 10^3^ M^−1^. While *I_0_*/*I* increased as phosphate concentrations increased, the ratio of lifetime (*τ_0_*/*τ*) was almost unchanged in the 0–100 μM phosphate concentrations in time-resolved photoluminescence experiments. On the contrary, both *I_0_*/*I* and *τ_0_*/*τ* increased as phosphate concentrations increased in the 100–500 μM phosphate concentrations (Supplementary Figure S4). This indicates the simultaneous existence of static and dynamic quenching phenomena [34]. The response time of the Eu-TCA dispersion was found to be 123 s from the time-dependent studies (Figure 3c).

**Figure 4:**
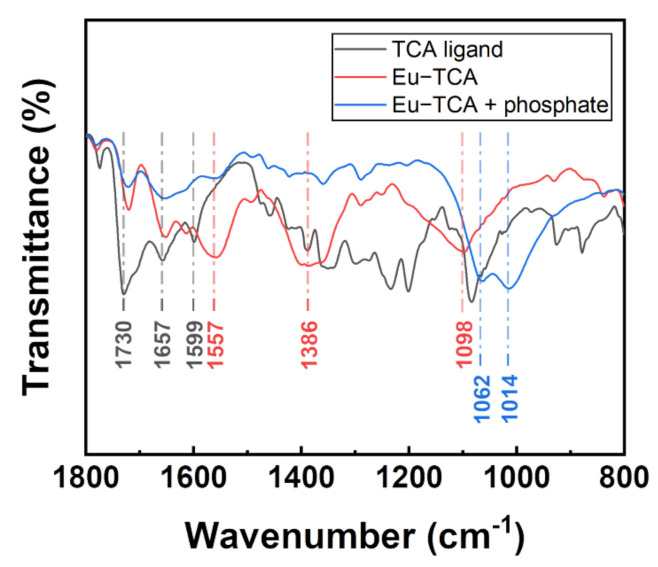
FTIR spectra of the TCA ligand and Eu-TCA before and after incubation with phosphate.

As shown in Figure 4, the formation of the Eu-TCA and its phosphate-binding event was monitored by using FTIR. The TCA ligand exhibited a strong C=O stretching peak at 1730 cm^−1^, which was red-shifted to 1720 cm^−1^ after the formation of the Eu-TCA. The asymmetric 1599 cm^−1^ peak from the carboxylic groups grew bigger and red-shifted to 1557 cm^−1^. The CH_2_ stretching vibration at 1386 cm^−1^ and the imide ring deformation peak at 1098 cm^−1^ increased due to the formation of coordination polymers with PVP. The carbonyl peak at 1657 cm^−1^ overlapped with the imide carbonyl peak, which originated from PVP. After incubation with phosphate, the intensities of the peaks at 1386 and 1098 cm^−1^ significantly decreased, indicating that the features of PVP considerably diminished. Two new peaks appeared at 1062 and 1014 cm^−1^. These corresponded to coordinated P–O bonds in two different binding modes [35]. Overall changes in the FTIR data strongly supported the binding of phosphate and rearrangement of coordination bonds around Eu^3+^ metal centers included in Eu-TCAs.

**Figure 5:**
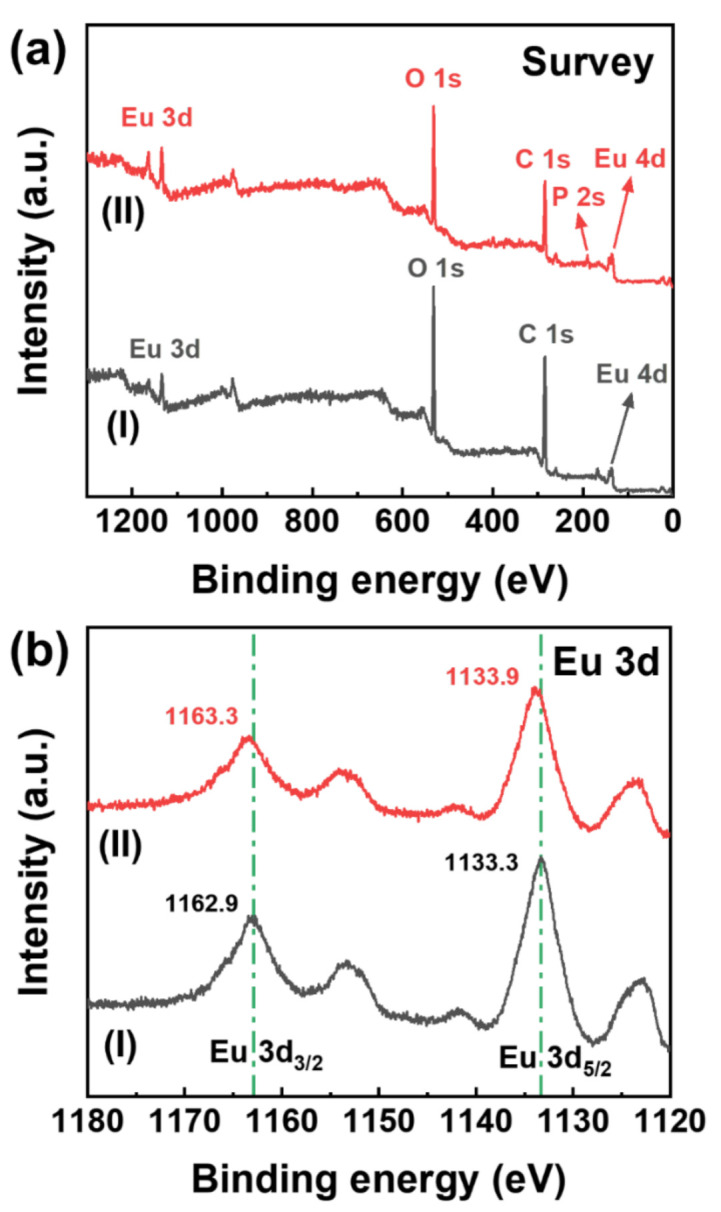
XPS spectra of Eu-TCA: (**a**) survey spectra of Eu-TCA before (I) and after (II) incubation with phosphate. (**b**) Eu 3d XPS spectra before (I) and after (II) incubation with phosphate.

XPS analysis was conducted to further investigate the coordination interactions between the Eu-TCA and phosphate. As shown in Figure 5a, the characteristic peaks corresponding to Eu, O, and C are observed from the Eu-TCA before and after incubation with phosphate. The peak at 190.1 eV, assigned to P 2s, indicates that phosphate ions are successfully introduced into the Eu-TCA as shown in Figure 5a (curve II). Figure 5b shows the Eu 3d spectra of the Eu-TCA before and after incubation with phosphate. The binding energies at 1162.9 and 1133.3 eV in Figure 5b (curve I) are assigned to Eu 3d_3/2_ and Eu 3d_5/2_, respectively, originating from the Eu-O clusters coordinated with carboxylic groups in the TCA ligands of the Eu-TCA. Similar to previous reports, the peaks of Eu 3d_3/2_ and Eu 3d_5/2_ were shifted toward higher binding energies after the inclusion of phosphate into the Eu-TCA framework as shown in Figure 5b (curve II) [24,36]. The shifts in the binding energy were caused by replacing carboxylic groups coordinated with Eu-O clusters with phosphoric groups. The interaction between Eu^3+^ ions and more electronegative P–O bonds resulted in the loss of electron density in Eu. Consequently, the binding energies of electrons in Eu 3d increased. The O 1s spectrum of the Eu-TCA was deconvoluted into four components positioned at 532.6, 531.8, 531.1, and 529.6 eV (Figure S5a). The peak at 529.6 eV represented lattice O bound to Eu (Eu-O-Eu). The other three peaks were related to water molecules or hydroxyl functional groups on the sample: the OH group with oxygen at the bridging oxygen site (bridging OH, 531.1 eV), the OH group with oxygen attached to Eu forming Eu-OH groups (terminal OH, 531.8 eV), and adsorbed water molecules on the sample surface (532.6 eV). After interacting with phosphate, the ratio of the components positioned at 532.6 and 531.8 eV to O 1s spectrum increased from 15.4% and 30.3% to 18.7% and 40.7%, respectively, whereas the ratio of the components peaked at 531.1 and 529.6 eV to O 1s spectrum decreased from 47.1% and 7.2% to 35.9% and 4.7%, respectively (Figure S5a,b). This phenomenon can be attributed to the formation of Eu–O–P bonds and the replacement of OH groups by phosphate [36]; this is consistent with the results of the FTIR analysis presented in Figure 4.

### 3.3. Phosphate Sensing Properties of Eu-TCA/GMF

**Figure 6:**
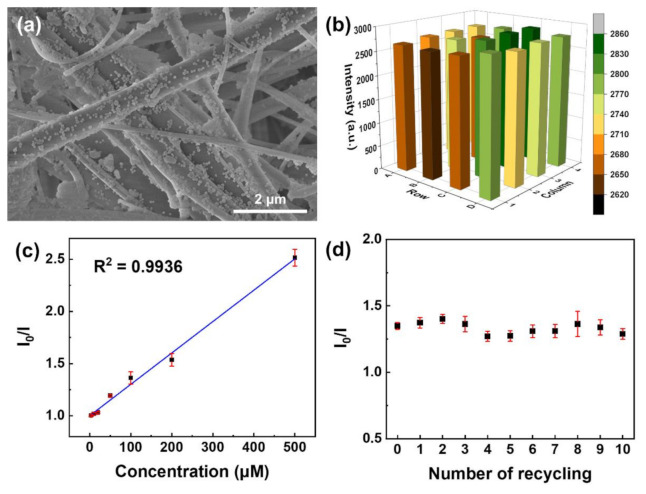
(**a**) SEM image of Eu-TCA/GMF. (**b**) Luminescence intensity of 4 × 4 wells of microplate containing the independently fabricated Eu-TCA/GMFs. (**c**) Stern–Volmer plot of the luminescence quenching of Eu-TCA/GMF (*n* = 7) as a function of phosphate concentration. (**d**) The luminescence intensity of Eu-TCA/GMF (*n* = 7) after ten runs of recycling. The concentration of phosphate is 100 μM.

For practical use, the sensing materials are recommended to be embedded in solid substrates because the recycling process of suspension in which the sensing material is homogeneously dispersed is complex and time-consuming. In this study, we propose the Eu-TCA/GMF as a reusable phosphate sensor. As shown in Figure 6a, the Eu-TCA nanoparticles are densely embedded on the surface of the microfiber over the entire filter, despite the washing process described in Materials and Methods. In order to evaluate the batch-to-batch reproducibility of the Eu-TCA/GMF, the luminescence intensities of the independently fabricated Eu-TCA/GMFs were investigated as shown in Figure 6b. The emission at 615 nm was monitored at an excitation wavelength of 260 nm, similar to the experiments in the Eu-TCA dispersion. The relative standard deviation of the luminescence intensity was 2.5%, which indicates that the batch-to-batch fabrication of the Eu-TCA/GMF is reproducible. 

To measure the luminescence intensity of the Eu-TCA/GMF under exposure to phosphate anions, the wells of the microplate containing the Eu-TCA/GMF were filled with 200 μL of phosphate solutions at concentrations ranging from 0 to 500 μM. Figure 6c shows *I_0_/I* as a function of phosphate concentration, where *I* and *I_0_* denote the luminescence intensities of the Eu-TCA/GMF in deionized water (200 μL) with and without phosphate, respectively. Upon the addition of phosphate to the wells of the microplate, the luminescence intensity of the Eu-TCA/GMF decreased. In addition, the phosphate detection of the Eu-TCA/GMF showed a linear response from 3 to 500 μM (R^2^ = 0.9936) with a *K*_SV_ of 3.01 × 10^3^ M^−1^. A short response time (123 s) was observed from the Eu-TCA dispersion, whereas the response times of the Eu-TCA/GMF were 982 s at 100 μM (Figure 3c and Supplementary Figure S6). The slow response of the Eu-TCA/GMF can be attributed to the limited effective surface and low reaction probability of Eu-TCA for the interaction with phosphate in the membrane. The limit of detection (LOD) was derived from the 3*σ* IUPAC criteria using the following equation:

(2) LOD=3σ/k ,

where *σ* is the standard deviation and *k* is the slope [37,38]. The standard deviation was calculated by using multiple luminescence intensities of the Eu-TCA/GMF in the absence of phosphate (blank). The slope was taken from the linear fit curve for luminescence intensity against phosphate concentration as shown in Figure S7. The LOD of the Eu-TCA/GMF was determined to be 1.52 μM, which is below the indicative level (3.23 μM) for probable problematic algal growth [14]. As shown in Supplementary Table S1, the sensing performance of the Eu-TCA/GMF was comparable or better than those of the other methods for phosphate detection in terms of LOD.

To study the performance of reused Eu-TCA/GMF, the wells of a microplate containing the Eu-TCA/GMF were incubated with an aqueous phosphate solution of 100 μM for complete interaction of phosphate with the Eu-TCA. Then, the microplate was washed several times with deionized water. After ten runs of the recycling process, *I_0_/I* barely changed, indicating that the Eu-TCA/GMF can act as a reusable sensing material with high repeatability for detecting phosphate (Figure 6d).

**Figure 7:**
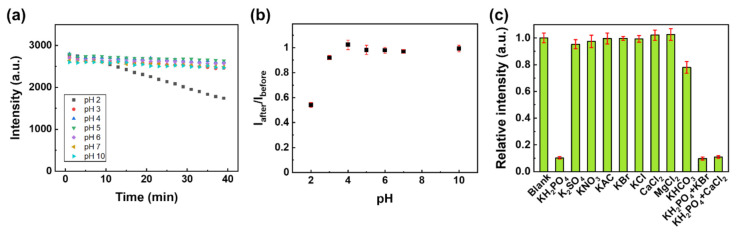
(**a**) The luminescence intensity of Eu-TCA/GMF as a function of time under various pH conditions. (**b**) *I*_after_/*I*_before_ of Eu-TCA/GMF (*n* = 6) as a function of pH, where *I*_before_ is the luminescence intensity before exposure to the pH solution and *I*_after_ is the luminescence intensity after exposure to the pH solution. (**c**) The luminescence intensity of Eu-TCA/GMF (*n* = 6) immersed in phosphate and other various ionic solutions (1 mM).

The effects of pH on the luminescence intensity of the Eu-TCA/GMF were studied. The wells of the microplate containing the Eu-TCA/GMF were filled with 200 μL of solutions in the pH range of 2–10. Figure 7a shows the luminescence intensity of the Eu-TCA/GMF under various pH conditions as a function of time. The luminescence intensity of the Eu-TCA/GMF at pH 2 showed a time-dependent decrease, whereas the luminescence intensity in the pH range of 3–10 was stable over time (40 min). To explore the recovery of the luminescence intensity, the Eu-TCA/GMF was washed with deionized water, and the luminescence intensity was measured with 200 μL of a pH 7 solution. Figure 7b shows *I*_after_/*I*_before_ of the Eu-TCA/GMF, where *I*_before_ is the luminescence intensity at pH 7 before exposure to the pH solution and *I*_after_ is the luminescence intensity at pH 7 after exposure to the pH solution. Except for *I*_after_/*I*_before_ of the Eu-TCA/GMF exposed to a pH 2 solution, the luminescent intensity was almost unchanged in the pH range of 3–10, suggesting that the Eu-TCA/GMF can be used in a wide range of pH conditions for detecting phosphate. In the case of other fluorescence-based phosphate sensing materials, a stable signal in such a wide pH range is not achieved (Supplementary Table S1). In this study, it is judged that pH stability was secured by forming a more stable CPP with the introduction of multidentate ligand containing three carboxylate functional groups.

Selective detection is crucial for applying phosphate monitoring in aquatic ecosystems, where various types of ions exist. To study the selectivity of the Eu-TCA toward phosphate, the luminescence intensity of the Eu-TCA/GMF was compared with various ions, including SO_4_^2−^, NO_3_^−^, CH_3_COO^−^, Br^−^, Cl^−^, HCO_3_^−^, K^+^, Mg^2+^, and Ca^2+^, which usually exist in aquatic environments. Figure 7c shows the luminescence intensity upon exposure to various anion solutions (1 mM). The addition of phosphate resulted in a drastic luminescence quenching effect. Except that the relative intensity in the presence of HCO_3_^−^ ion was 0.8, no obvious change in luminescence intensity was observed for the other ions. The effects of coexisting ions on the luminescence intensity were also investigated. The molar concentrations of phosphate and interfering analytes (KBr or CaCl_2_) were 1 mM and 3 mM, respectively. Although the concentration of interfering analyte is three times higher, the detection of phosphate was not affected by other ions, indicating an exceptional recognition capability of the Eu-TCA for phosphate in an aqueous solution. 

To investigate the reliability of the Eu-TCA/GMF in practical applications, the phosphate detection was tested in tap water and lake water from Daecheong Lake as representative real environmental samples. The water samples were spiked with known concentrations of the phosphate solution. As summarized in Table 1, the recoveries and the corresponding relative standard deviation values of the spiked samples were found in the range of 95–104% and 2–4%, respectively, suggesting that the proposed sensing platform is feasible for the phosphate detection in real environmental samples.

## 4. Conclusions

This study demonstrates that luminescent nanoparticles (Eu-TCAs) composed of europium and multidentate ligands can be synthesized as CPPs that respond to phosphate through specific coordination interactions between lanthanides and phosphates, as confirmed by IR spectroscopy and XPS analysis. We considered that selective interactions between europium and phosphates provide exceptional selectivity and sensitivity in the application of phosphate sensors, and CPP instead of crystalline metal–organic frameworks promotes chemical stability under various pH conditions, from acidic to basic environments, with the aid of PVPs as a coat, capping, and matrix material. The nanoparticles were embedded into the glass microfiber filter, and exhibited a detection limit of 1.52 μM. Furthermore, the Eu-TCA/GMF showed durability from pH 3 to pH 10 and selectivity to phosphate ion against potential interfering ions in aqueous environment. Based on this study, a membrane containing luminescent nanoparticles is expected to be installed in a probe-type photoluminescent sensor and operated without any supply of redox chemicals or any activation chemical process.

## Figures and Tables

**Table 1:** Comparison of phosphate detection in real water samples using the proposed Eu-TCA/GMF.

Samples	Spiked (μM)	Measured (μM) (Mean ± Standard Deviation, *n* = 3)	Recovery (%) (Mean ± Standard Deviation, *n* = 3)
Tap water	0	not detected ^1^	
	20	20.82 ± 0.58	104.10 ± 2.90
	50	50.30 ± 1.22	100.60 ± 2.44
Lake water (Daecheong Lake)	0	not detected ^1^	
	20	19.77 ± 0.46	98.85 ± 2.30
	50	47.45 ± 2.06	94.90 ± 4.12

^1^ The determination of phosphate in unspiked samples was also confirmed by phosphomolybdenum blue spectrophotometric methods.

## Data Availability

Not applicable.

## Supplementary Materials

The following are available online at https://www.mdpi.com/article/10.3390/polym14010190/s1, Supplementary Figure S1: SEM image of Eu-TCA microcrystals, Supplementary Figure S2: Dynamic light scattering analysis of Eu-TCA, Supplementary Figure S3: Emission spectrum of Eu-TCA dispersion (λ_ex_ = 260 nm). The inset shows enlarged emission spectrum, Supplementary Figure S4: Stern–Volmer plot for the luminescence quenching of Eu-TCA dispersion (*n* = 5) upon addition of different concentrations of phosphate. Inset shows the Stern–Volmer plot in the concentration range of 2–500 μM, Supplementary Figure S5: Experimental fit performed on O 1s XPS spectra before (a) and after (b) incubation with phosphate, Supplementary Figure S6: Time-dependent luminescence intensity of the Eu-TCA/GMF upon the addition of 100 μM of phosphate, Figure S7: The luminescence intensity of Eu-TCA/GMF (*n* = 7) under various phosphate concentrations and their linear fit curve for the estimation of LOD, Table S1: Comparison of various analytical methods for phosphate detection.
