# Supplementary material for: Reusable and pH-Stable Luminescent Sensors for Highly Selective Detection of Phosphate
Source: Polymers (Basel). 2022 Jan 4;14(1):190. doi: 10.3390/polym14010190 (PMC8747124; doi:10.3390/polym14010190)
Supplement: Supplementary file 1 [file polymers-14-00190-s001.zip › polymers-1514234-supplementary.pdf]

Supporting Information

Reusable and pH-Stable Luminescent Sensors for Highly Selective Detection of Phosphate

Do Yeob Kim [1,†], Dong Gyu Kim [2,†], Bongjin Jeong [1], Young Il Kim [2], Jungseok Heo [2,*] and Hyung-Kun Lee [1,*]

1 ICT Creative Research Laboratory, Electronics & Telecommunications Research Institute, Daejeon 34129, Korea; nanodykim@etri.re.kr (D.Y.K.); jbj0919@etri.re.kr (B.J.)
2 Department of Chemistry, Chungnam National University, Daejeon 34134, Korea; kdg05262@naver.com (D.G.K.); yi4902@naver.com (Y.I.K.)
* Correspondence: jungseokheo@cnu.ac.kr (J.H.); hklee@etri.re.kr (H.-K.L.); Tel.: +82-42-860-5857 (H.-K.L.)
† These authors contributed equally to this work.

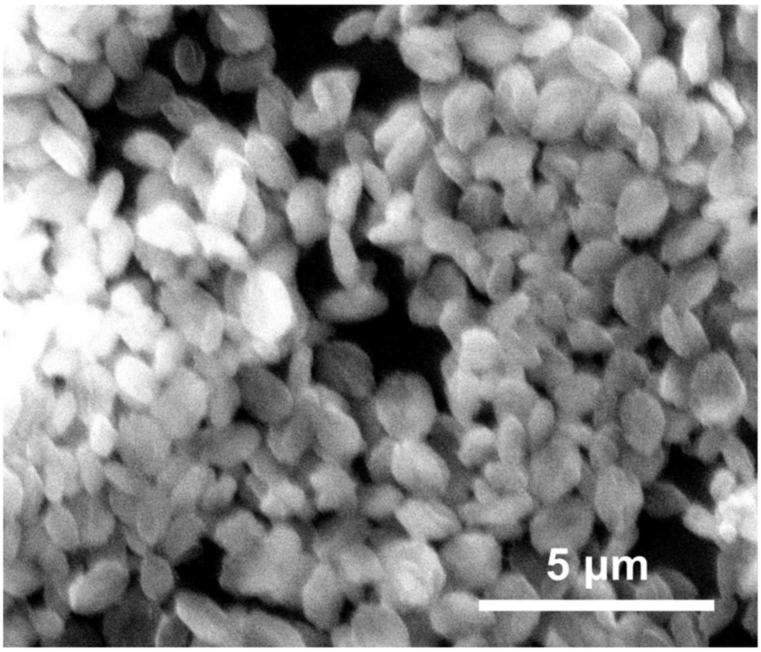

Figure S1. SEM image of Eu-TCA microcrystals.

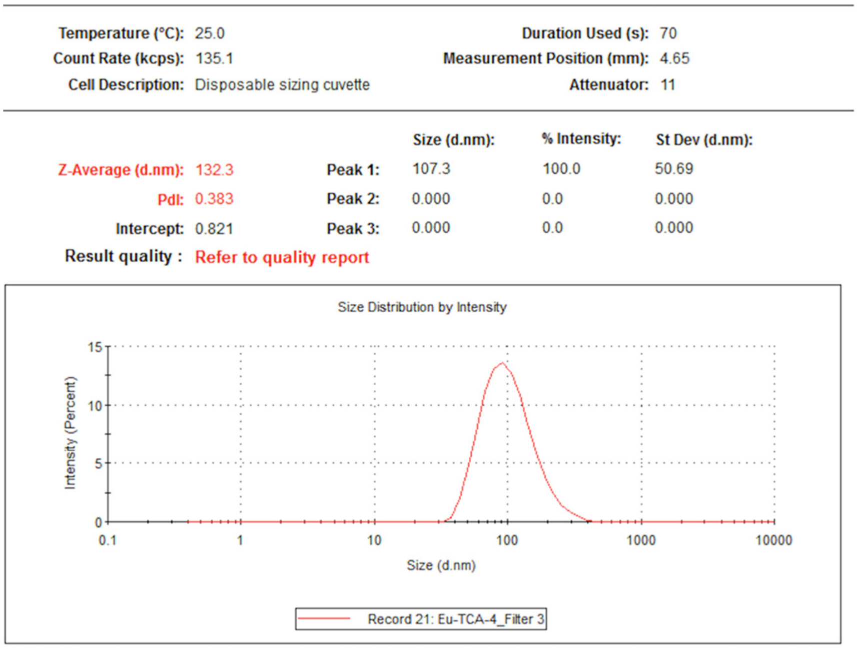

Figure S2. Dynamic light scattering analysis of Eu-TCA.

Synthesis of Eu-TCA microcrystals

$EuCl_3$ (235 mg, 0.64 mmol, 5.3 eq.), TCA ligand (60 mg, 0.12 mmol, 1 eq.), and polyvinylpyrrolidone (533 mg) were reacted under solvothermal conditions in a mixed solvent of dimethylformamide (32 mL) and ethanol (19 mL). The reaction was conducted at 150°C for 12 h. After the reaction, the reaction mixture was cooled to room temperature over the next 6 h, and colloidal particles were precipitated by centrifugation. Three cycles of washing with dry dimethylformamide and centrifugation were followed by a final wash with ethanol. The sample was dried in an oven at 70°C for 3 h.

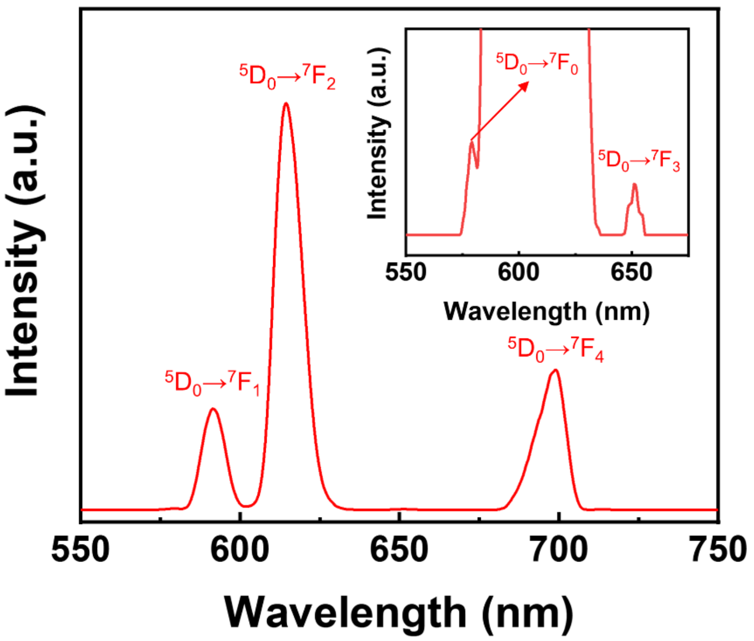

Figure S3. Emission spectrum of Eu-TCA dispersion (λ_{ex} = 260 nm). The inset shows enlarged emission spectrum.

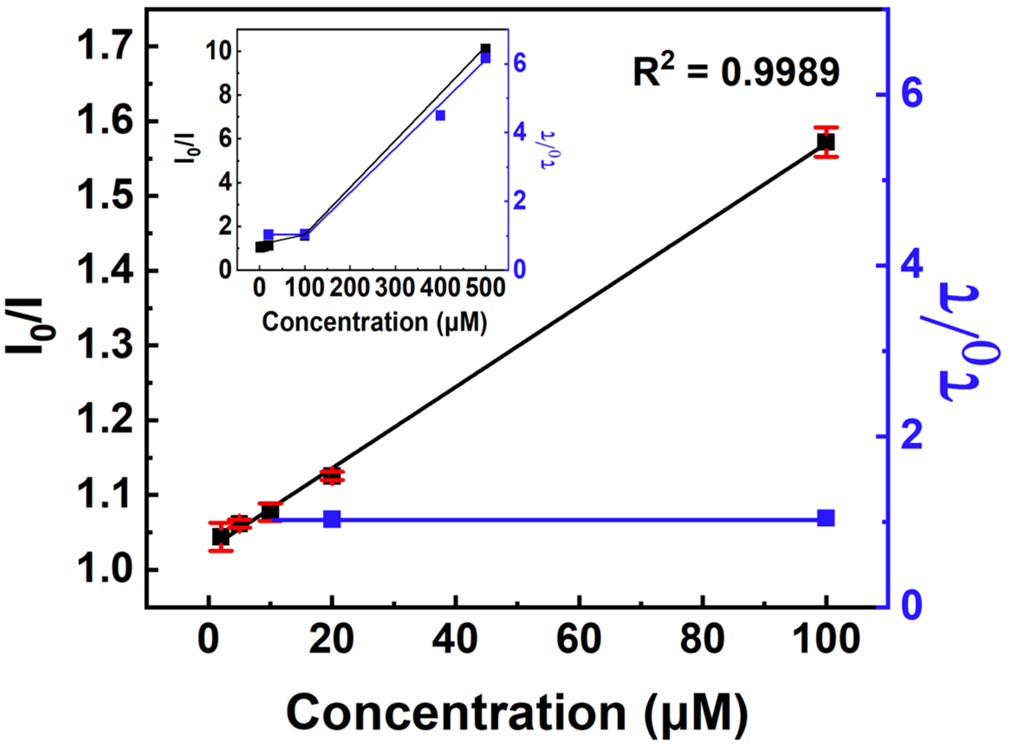

Figure S4. *Stern-Volmer* plot for the luminescence quenching of Eu-TCA dispersion upon addition of different concentrations of phosphate. Inset shows the *Stern-Volmer* plot in the concentration range of 2–500 μM.

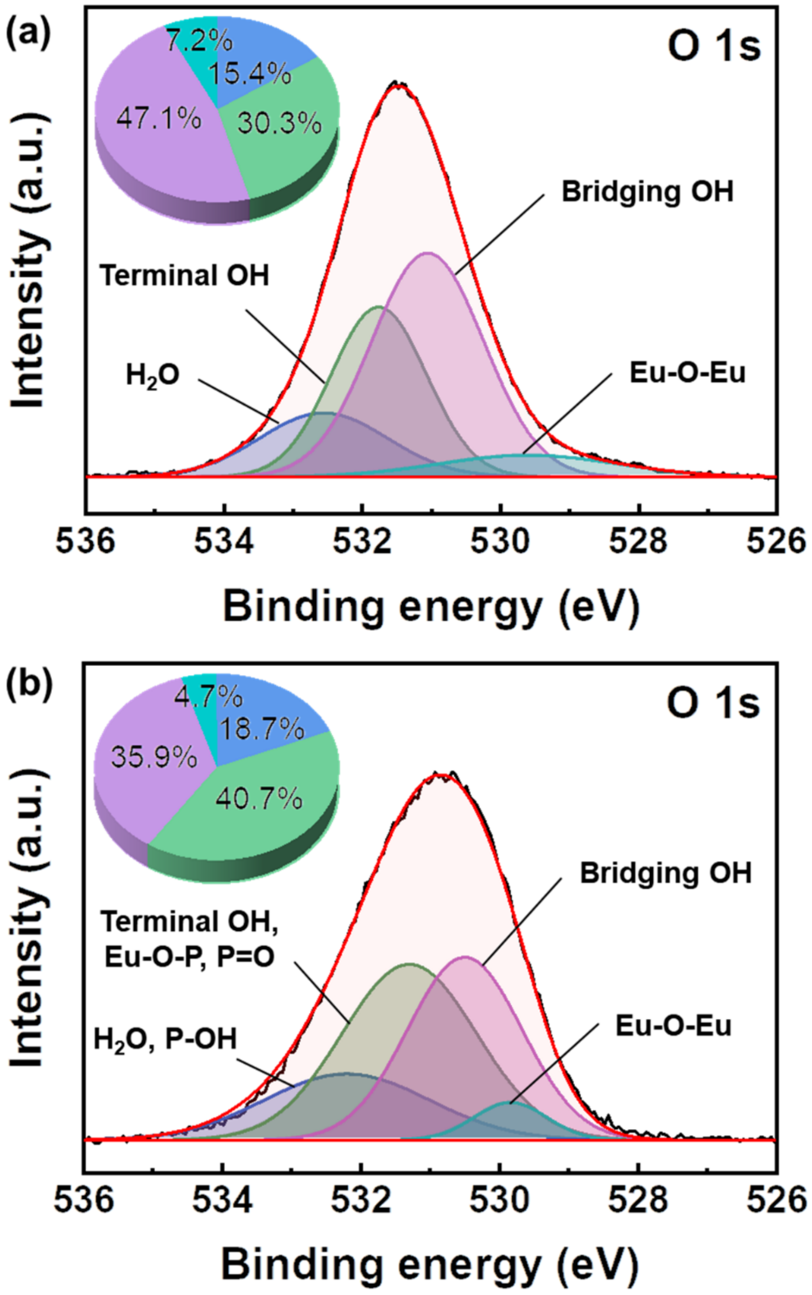

Figure S5. Experimental fit performed on O 1s XPS spectra before (a) and after (b) incubation with phosphate.

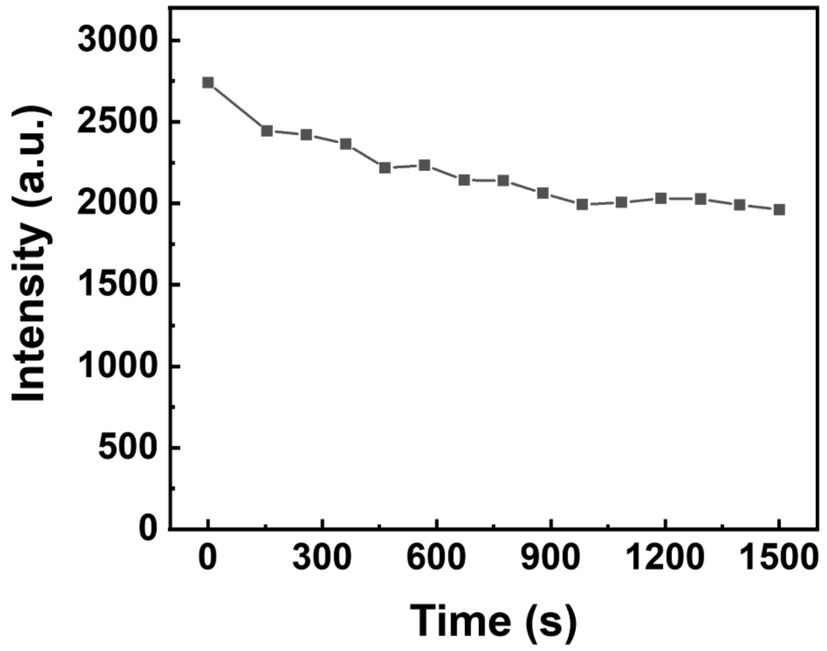

Figure S6. Time-dependent luminescence intensity of the Eu-TCA/GMF upon the addition of 100 μM of phosphate.

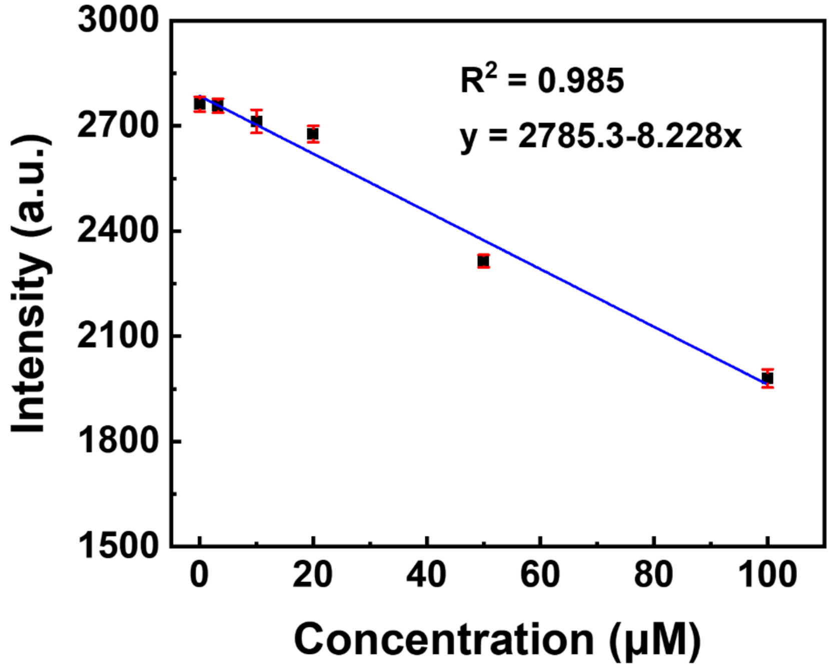

Figure S7. The luminescence intensity of Eu-TCA/GMF under various phosphate concentrations and their linear fit curve for the estimation of LOD.

Table S1. Comparison of various analytical methods for phosphate detection.

Analytical methods	Sensing element/structure	Linear range (μM)	LOD (μM)	Reusable [1]	pH range [2]	Ref.
Potentiometry	Mo-based electrode	$10–10^5$	1.9	O	pH-unstable [3]	S1
	W-based electrode	$1–10^5$	0.4	O	pH-unstable [3]	S2
	Polymeric membrane electrode	$32–10^5$	10	O	pH-unstable [3]	S3
Voltammetry	Screen-printed electrodes modified with carbon black nanoparticles	0.5–100	0.1	O	pH-unstable [3]	S4
I-V measurement	Ag/graphene composite-based field-effect transistor	5–6000	1.2	O	–	S5
Colorimetric probe	Au nanoparticles/Eu^{3+}	0.5–30	0.076	X	–	S6
	Functionalized Au nanoparticles	80–200	120	X	7–8	S7
Luminescent probe	Eu@BUC-14	5–150	0.88	X	4–8	S8
	UiO-66-NH_2 MOF [4]	5–150	1.25	X	–	S9
	Eu-triazole MOF [4]	3–30	6.62	X	–	S10
	Tb-MOF [4]	40–400	35	X	–	S11
	Eu-based nanospheres	2–100	0.83	X	5–9	S12
	Eu-TCA/GMF	3–500	1.52	O	3–10	This work

[1] The term "reusable" means the sensing material can be easily reused without complex processes such as centrifugation or drying. [2] pH range that does not affect the sensor performance. [3] Needs pH compensation model. [4] MOF: metal-organic framework.

References

1. Li, Y.; Jiang, T.; Yu, X.; Yang, H. Phosphate Sensor Using Molybdenum. *J. Electrochem. Soc.* **2016**, *163*, B479–B484.

2. Chen, G.; Xiao, S.; Lorke, A.; Liu, J.; Zhang, P. Assessment of a Solid-State Phosphate Selective Electrode Based on Tungsten. *J. Electrochem. Soc.* **2018**, *165*, B787–B794.

3. Kim, J.; Kang, D. M.; Shin, S. C.; Choi, M. Y.; Kim, J.; Lee, S. S.; Kim, J. S. Functional Polyterthiophene-Appended Uranyl-Salophen Complex: Electropolymerization and Ion-Selective Response for Monohydrogen Phosphate. *Analytica Chimica Acta* **2008**, *614*, 85–92.

4. Talarico, D.; Arduini, F.; Amine, A.; Moscone, D.; Palleschi, G. Screen-Printed Electrode Modified with Carbon Black Nanoparticles for Phosphate Detection by Measuring the Electroactive Phosphomolybdate Complex. *Talanta* **2015**, *141*, 267–272.

5. Bhat, K. S.; Nakate, U. T.; Yoo, J.-Y.; Wang, Y.; Mahmoudi, T.; Hahn, Y.-B. Nozzle-Jet-Printed Silver/Graphene Composite-Based Field-Ef fect Transistor Sensor for Phosphate Ion Detection. *ACS Omega* **2019**, *4*, 8373–8380.

6. Liu, W.; Du, Z.; Qian, Y.; Li, F. A Specific Colorimetric Probe for Phosphate Detection Based on Anti-Aggregation of Gold Nanoparticles. *Sensors and Actuators B: Chemical* **2013**, *176*, 927–931.

7. He, G.; Zhao, L.; Chen, K.; Liu, Y.; Zhu, H. Highly Selective and Sensitive Gold Nanoparticle-Based Colorimetric Assay for PO_4^{3-} in Aqueous Solution. *Talanta* **2013**, *106*, 73–78.

8. Zhang, Y.; Sheng, S.; Mao, S.; Wu, X.; Li, Z.; Tao, W.; Jenkinson, I. R. Highly Sensitive and Selective Fluorescent Detection of Phosphate in Water Environment by a Functionalized Coordination Polymer. *Water Research* **2019**, *163*, 114883.

9. Yang, J.; Dai, Y.; Zhu, X.; Wang, Z.; Li, Y.; Zhuang, Q.; Shi, J.; Gu, J. Metal-Organic Frameworks with Inherent Recognition Sites for Selective Phosphate Sensing through Their Coordination-Induced Fluorescence Enhancement Effect. *J. Mater. Chem. A* **2015**, *3*, 7445–7452.

10. Chandra Rao, P.; Mandal, S. Europium-Based Metal-Organic Framework as a Dual Luminescence Sensor for the Selective Detection of the Phosphate Anion and Fe^{3+} Ion in Aqueous Media. *Inorg. Chem.* **2018**, *57*, 11855–11858.

11. Asha, K. S.; Bhattacharjee, R.; Mandal, S. Complete Transmetalation in a Metal-Organic Framework by Metal Ion Metathesis in a Single Crystal for Selective Sensing of Phosphate Ions in Aqueous Media. *Angew. Chem. Int. Ed.* **2016**, *55*, 11528–11532.

12. Song, X.; Ma, Y.; Ge, X.; Zhou, H.; Wang, G.; Zhang, H.; Tang, X.; Zhang, Y. Europium-Based Infinite Coordination Polymer Nanospheres as an Effective Fluorescence Probe for Phosphate Sensing. *RSC Adv.* **2017**, *7*, 8661–8669.
